# Microstructural, mechanical, and electrochemical analysis of carbon doped AISI carbon steels

**DOI:** 10.1186/s42649-022-00079-w

**Published:** 2022-10-20

**Authors:** Muhammad Ishtiaq, Aqil Inam, Saurabh Tiwari, Jae Bok Seol

**Affiliations:** 1 Department of Materials Engineering and Convergence Technology, Gyeongsang National University (GNU), Jinju, 52828 Republic of Korea; 2 Department of Materials Engineering and Convergence Technology, Center for K-metal, Gyeongsang National University (GNU), Jinju, 52828 South Korea; 3 Institute of Metallurgy and Materials Engineering, University of the Punjab, Lahore, Pakistan

**Keywords:** Carbon steels, SEM, Electrochemical, Heat treatments, Corrosion rate

## Abstract

The effect of carbon doping contents on the microstructure, hardness, and corrosion properties of heat-treated AISI steel grades of plain carbon steel was investigated in this study. Various microstructures including coarse ferrite-pearlite, fine ferrite-pearlite, martensite, and bainite were developed by different heat treatments i.e. annealing, normalizing, quenching, and austempering, respectively. The developed microstructures, micro-hardness, and corrosion properties were investigated by a light optical microscope, scanning electron microscope, electromechanical (Vickers Hardness tester), and electrochemical (Gamry Potentiostat) equipment, respectively. The highest corrosion rates were observed in bainitic microstructures (2.68–12.12 mpy), whereas the lowest were found in the fine ferritic-pearlitic microstructures (1.57–6.36 mpy). A direct correlation has been observed between carbon concentration and corrosion rate, i.e. carbon content resulted in an increase in corrosion rate (2.37 mpy for AISI 1020 to 9.67 mpy for AISI 1050 in annealed condition).

## Introduction

Since steel has been the most important engineering material for long periods, mankind has an enormous experimental database on steel microstructures and mechanical properties (Kim et al. 2022; Kim et al. 2021a; Ko et al. 2022; Kim et al. 2021b). Especially, carbon-doped steels find their applications in marine, power plants, construction, processing equipment, etc. (Basak et al. 1998; Kuruvilla 1998; Uhling and Reive 1985). The steels specifically carbon steels are vulnerable to corrosion and it costs about 2.5 trillion USD ~ 3.4% of GDP globally every year according to the NACE impact report 2013 (Koch et al. 2013). The environment to which a material is exposed plays a vital role in its service life and performance. Moisture, rain, man-made solutions, and in particular seawater are the most prominent environments for corrosion (Kim et al. 2005a; Fontana 1986; Riazi et al. 2013).

The chemical compositions and heat-treatment routes of the steels are the prime features, capable of achieving artificial intelligence (AI)-driven alloy development. Modifying these two parameters determines the steel microstructures and associated mechanical performance for a given corrosive environment e.g., water, oxygen, or humid environment (Kermani and Morshed 2003; Nesic and Lunde 1994; Ueda and Takabe 1999; Nesic et al. 1996; Palacios and Shadley 1993; Dugstad et al. 2000; John and Sweet 1998; Waard et al. 1995; Gulbrandsen et al. 2000). Along-with steel composition (Kang et al. 2019), employing different heat-treatment processes is a facile route to improve not only the microstructure stability but also the corrosion properties (Cots et al. 2003). Hence, the systematic study on the relation between the two parameters and the microstructures is vital for the upcoming AI era.

Clover et al. (Clover et al. 2005) suggested that amongst the steels having 0.06–0.19 wt.% C, the sample comprising coarse ferrite and acicular pearlite, would be treated as superior microstructure to the material having banded ferrite-pearlite microstructure in terms of corrosion resistance. However, they also found that penetration rates were lower (3.9–5.2 mm y^− 1^) for low carbon steels (< 0.10% C) and higher (3.3–6.4 mm y^− 1^) for steels having higher carbon contents. Here, they only considered ferrite pearlite microstructures for corrosion analysis whereas in many applications martensitic or bainitic carbon steels are employed and carbon doping effects on these structures were yet to be explored. Similarly, Guo et al. (Guo et al. 2008a; Guo et al. 2008b) studied carbon steels having 0.03–0.1 wt.% C and found that microstructure formed with lower carbon content did not affect the corrosion rate of steels significantly but with an increase in the carbon content, considerable variation in corrosion rate was observed in single-phase bainitic steel in comparison to multi-phase steel containing ferrite and cementite phases. Here, although they studied the bainitic steels along-with ferritic-pearlitic steels, however, the carbon doping was limited to 0.1% only and they didn’t provide a comparison with martensitic structured steels. Pleshivtsev et al. (Pleshivtsev et al. 2009) reported that increase in carbon contents from 0.04–0.215 wt.% resulted in an increased corrosion rate of almost 2%. It has also been reported that microstructures containing ferrite-pearlite exhibited better corrosion resistance than tempered martensitic microstructures (Pleshivtsev et al. 2009). Although they compared ferritic-pearlitic structures with martensitic but still carbon doping range was 0.04–0.215 wt.%. There are some more studies on the importance of the microstructures and heat-treatments on the corrosion behavior of the steels (Schmitt and Horstemeier 2006; Takabe and Ueda 2001; Farelas et al. 2012). Despite such intensive attempts, the influence of carbon concentrations on the microstructure and associated corrosion resistance properties for plain carbon steels having exclusively only carbon over a wider range of 0.2 to 0.50 wt.% is still unclear. Plain carbon steels are important because they are the first-choice materials for pipeline designers due to their superior durability and recycling-ability as compared to other materials.

As the broader carbon doping effect has not been explored yet, this work aims to investigate the effects of carbon concentrations and microstructures on the electrochemical and mechanical behavior of different plain carbon steels (0.19 to 0.54 wt.% C). For this purpose, commonly used plain carbon steels i.e. AISI 1020,1030,1040,1045 and 1050, were selected, which were further processed by annealing, normalizing, quenching, and austempering heat-treatments. The electrochemical behavior of the heat-treated steel specimens was investigated in a 3.5% NaCl solution. Moreover, the morphology of the corroded surfaces, elemental composition, and dispersion of corrosion deposits were also analyzed. This manuscript provides detailed information about the effect of different microstructures, including ferrite, pearlite, bainite, and martensite, produced by different heat-treatment processes, on the corrosion resistance of a wide ranged (0.19–0.54 wt.%) carbon doped steels in NaCl solution.

## Materials and methods

### Materials

The plain carbon steel grades AISI 1020, 1030, 1040, 1045, and 1050 were acquired from Peoples Steel Mill Limited, Karachi, Pakistan in the form of hot-rolled bars. The chemical composition of these steels is given in Table 1. Samples of ~ 1cm^3^ size were, wire-cut from these bars for heat treatment and subsequent characterization.

**Table 1:** Chemical compositions (wt.%) of AISI grade plain carbon steels studied, determined by optical emission spectrometer *(MetaLab, Germany)*

AISI Steel Grade	C	Mn	Si	*P*	S
1020	0.19	0.49	0.24	0.015	0.023
1030	0.34	0.73	0.27	0.019	0.023
1040	0.37	0.82	0.26	0.013	0.023
1045	0.45	0.68	0.24	0.019	0.018
1050	0.54	0.79	0.34	0.016	0.030

### Heat-treatment

The test samples were cleaned in a 10% NaOH solution at 60 °C for 10 min followed by rinsing in water before starting the heat treatment. Cleaned samples were austenitized at 900, 870, 850, 840, and 820 °C for 30 minutes respectively. After austenitizing, the samples were furnace cooled for annealing, air-cooled for normalizing, and oil-cooled for quenching heat-treatments. For austempering, samples were salt-bath quenched between bainite start temperature (B_S_) and martensite start temperature (M_S_), held for 60 minutes, and then cooled in the air to room temperature. Corresponding austenitizing and austempering temperatures for experimental steels are given in Table 2. Bainite start (B_S_) temperature is calculated (Eq. 1) by J. S. Kirkaldy relation (Kang et al. 2014) for the austempering process.

1 $${\textrm{B}}_{\textrm{s}}=656-57.7\textrm{C}-75\textrm{Si}-35\textrm{Mn}-15.3\textrm{Ni}-34\textrm{Cr}-41.2\textrm{Mo}$$

**Table 2:** Parameters of heat treatment processes applied to experimental plain carbon steels

AISI Steel Grade	Austenitizing Temp. (°C)	Austempering Temp. B_s_ (°C)	Martensitic start Temp. M_s_ (°C)
1020	900	520	420
1030	870	495	363
1040	850	450	352
1045	840	395	331
1050	820	370	300

### Metallography

**Fig. 1:**
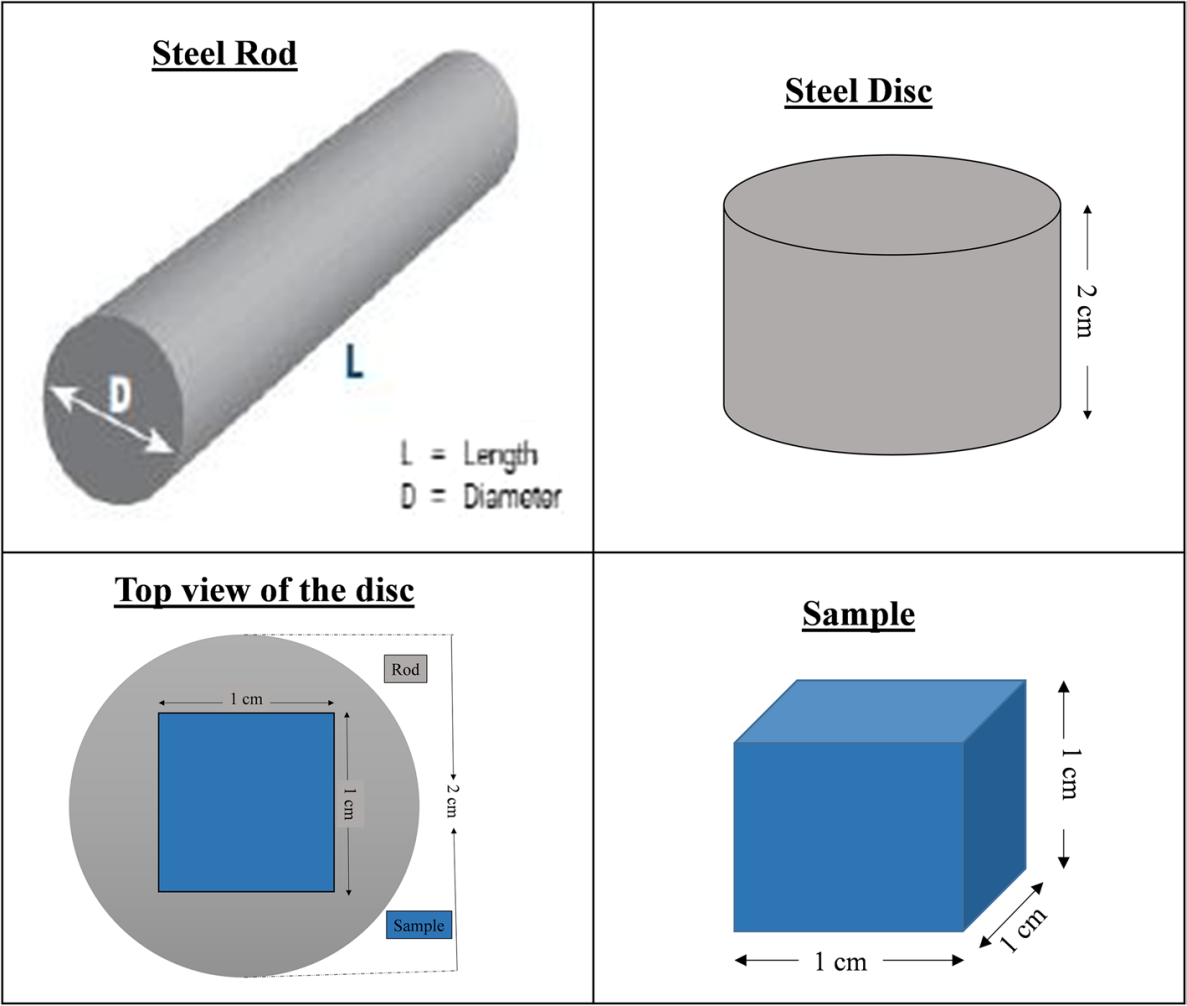
Schematic representation of sample cutting from rod

Slices were cut from the rod and samples of dimensions ~ 1 cm^3^ were cut from the middle of the slice as shown in schematic Fig. 1.

After cutting, all the samples were annealed to make them free from residual stresses and to get uniform microstructure prior to further heat treatment. Metallographic samples were prepared by standard practice and procedures. Polishing was done on a Buehler brand automatic polisher. Rough polishing was performed using Nylon cloth using the diamond pastes of 6 and 3 μm grit. Fine polishing was performed on velvet cloth with a diamond paste of 1 and 0.25 μm grit according to the ASTM E3 standard. The microstructure was observed from top of the disc (longitudinal plane i.e. parallel to the rolling direction).

### Hardness testing

Micro-Vickers hardness tester (Shimadzu brand) having a diamond indenter of size 1/16 in. was used for hardness testing. An average of five readings of hardness values was taken for the reliability.

### Electrochemical analysis

Cold mounted steel samples with dimensions of ~ 1 cm^3^, connected to a single copper wire by soldering, were used for electrochemical testing. The samples were prepared metallographically using different grit size SiC papers i.e., P200, P400, P800, and P1000. Three-electrode cell system with 3.5% NaCl solution was employed for electrochemical testing at room temperature. Saturated Calomel (250 mV) vs. standard hydrogen electrode (SHE) was used as a reference electrode, solid graphite rod, and heat-treated steel sample were used as an auxiliary electrode and working electrode respectively. These electrodes were connected to a Potentiostat (Gamry 5000P USA) via a cell cable.

### Surface analysis of steel sample after electrochemical testing

After Tafel polarization scans of heat-treated plain carbon steels in 3.5% NaCl, the corroded surfaces were analyzed under a scanning electron microscope (FEI Inspect S50SEM) at 600X, 1500X, and 10,000X. Elemental compositions of corrosion products were analysed using an energy dispersive x-ray spectrometer (EDS) attached to SEM by spot analysis and elemental mapping.

## Results and discussion

### Microstructure evolution

**Fig. 2:**
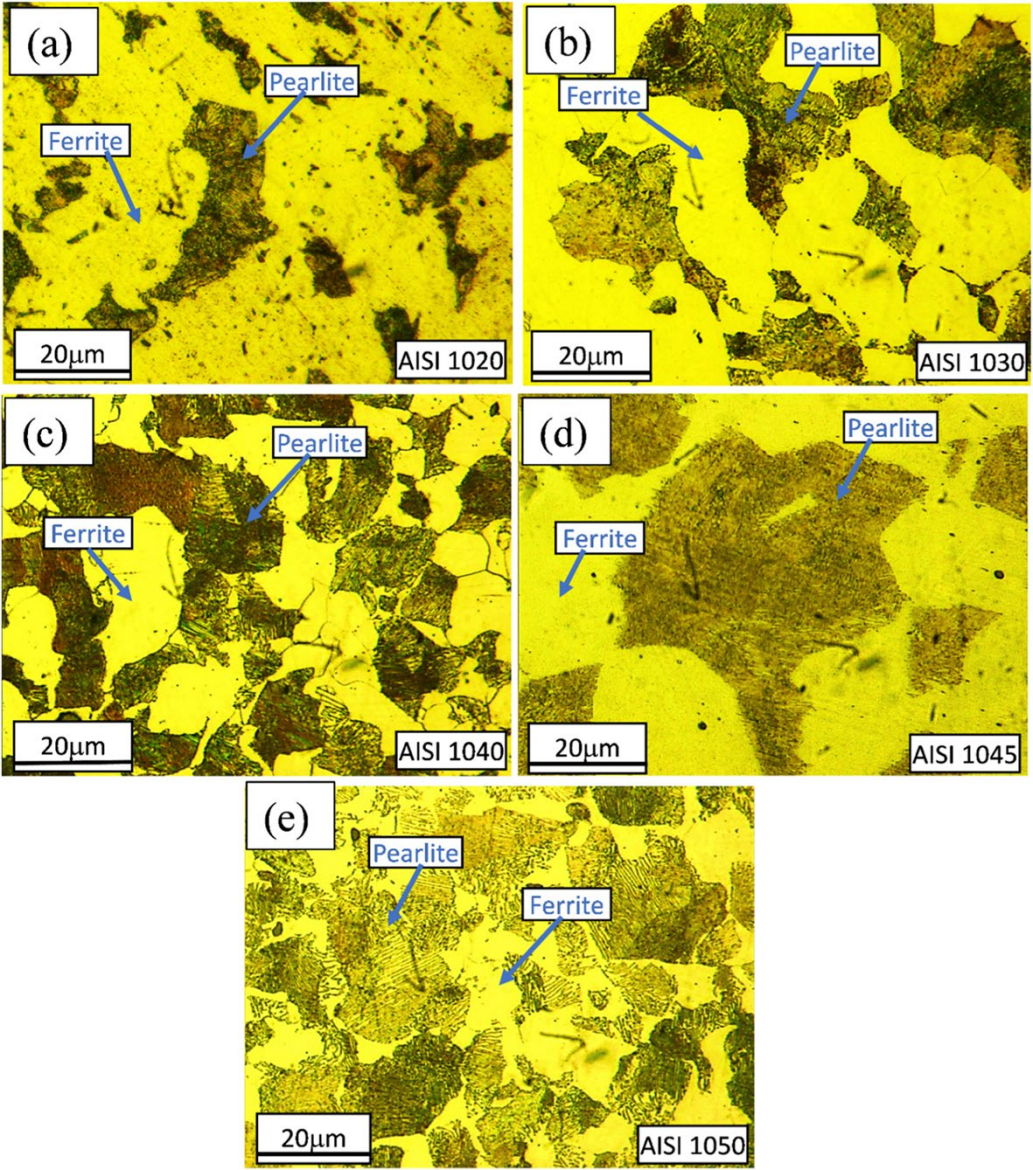
Light optical micrographs of AISI **a** 1020, **b** 1030, **c** 1040, **d** 1045, and **e** 1050 plain carbon steels obtained after annealing showing coarse ferritic-pearlitic microstructures

**Fig. 3:**
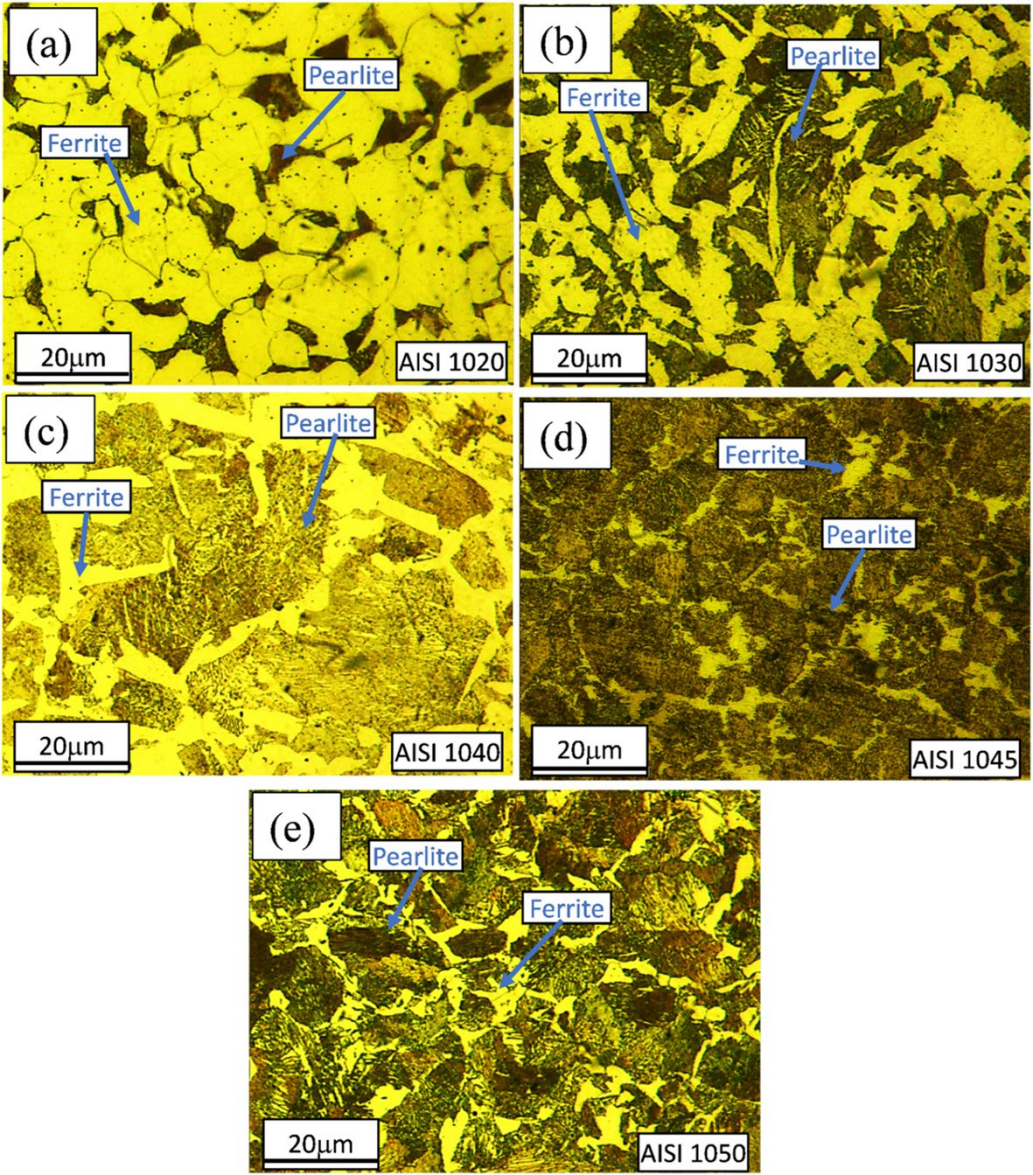
Light optical micrographs of AISI **a** 1020, **b** 1030, **c** 1040, **d** 1045 and **e** 1050 plain carbon steels obtained after normalizing showing fine ferritic-pearlitic microstructures

**Fig. 4:**
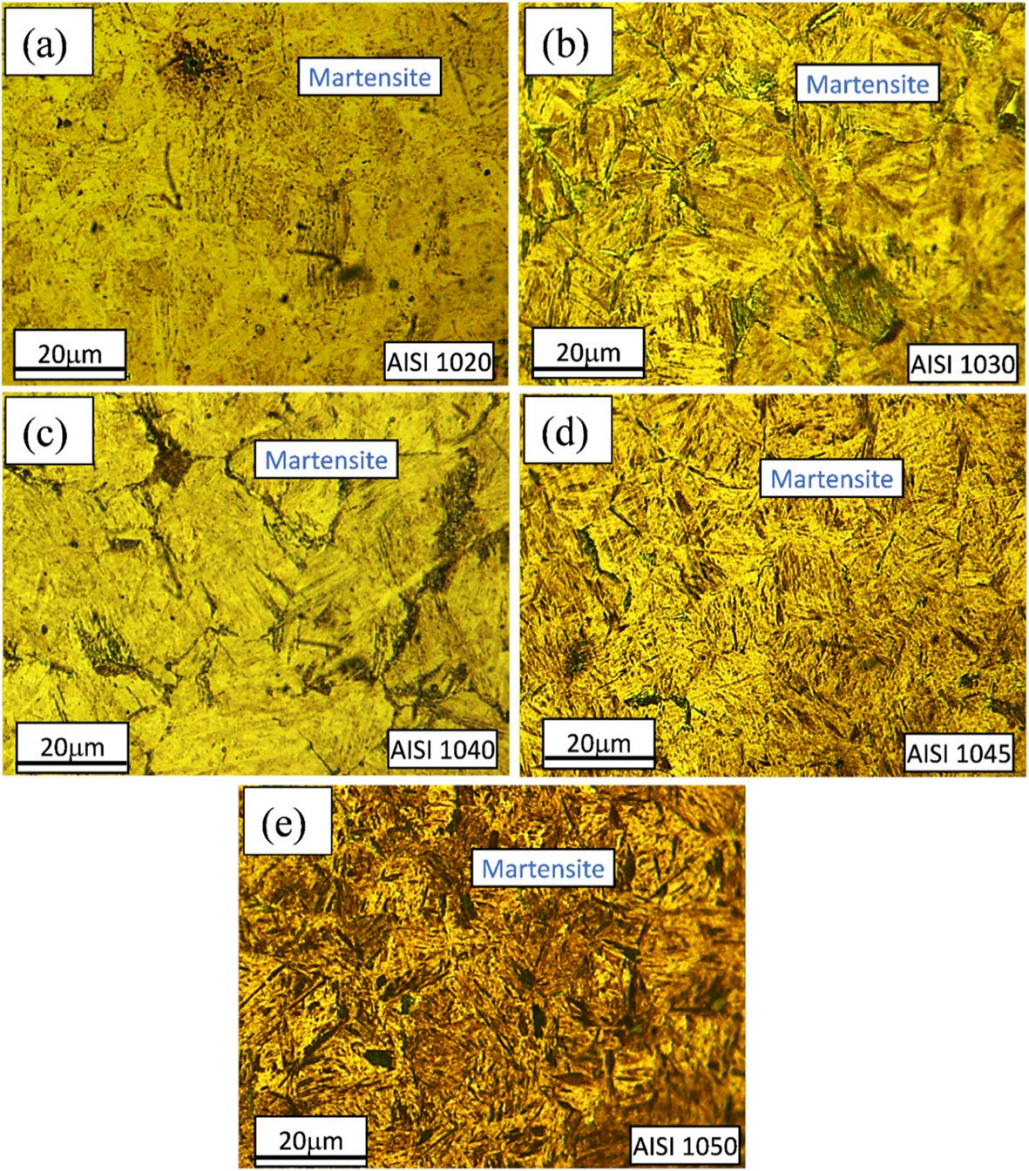
Light Optical micrographs of AISI **a** 1020, **b** 1030, **c** 1040, **d** 1045 and **e** 1050 plain carbon steels obtained after quenching showing martensitic microstructure

**Fig. 5:**
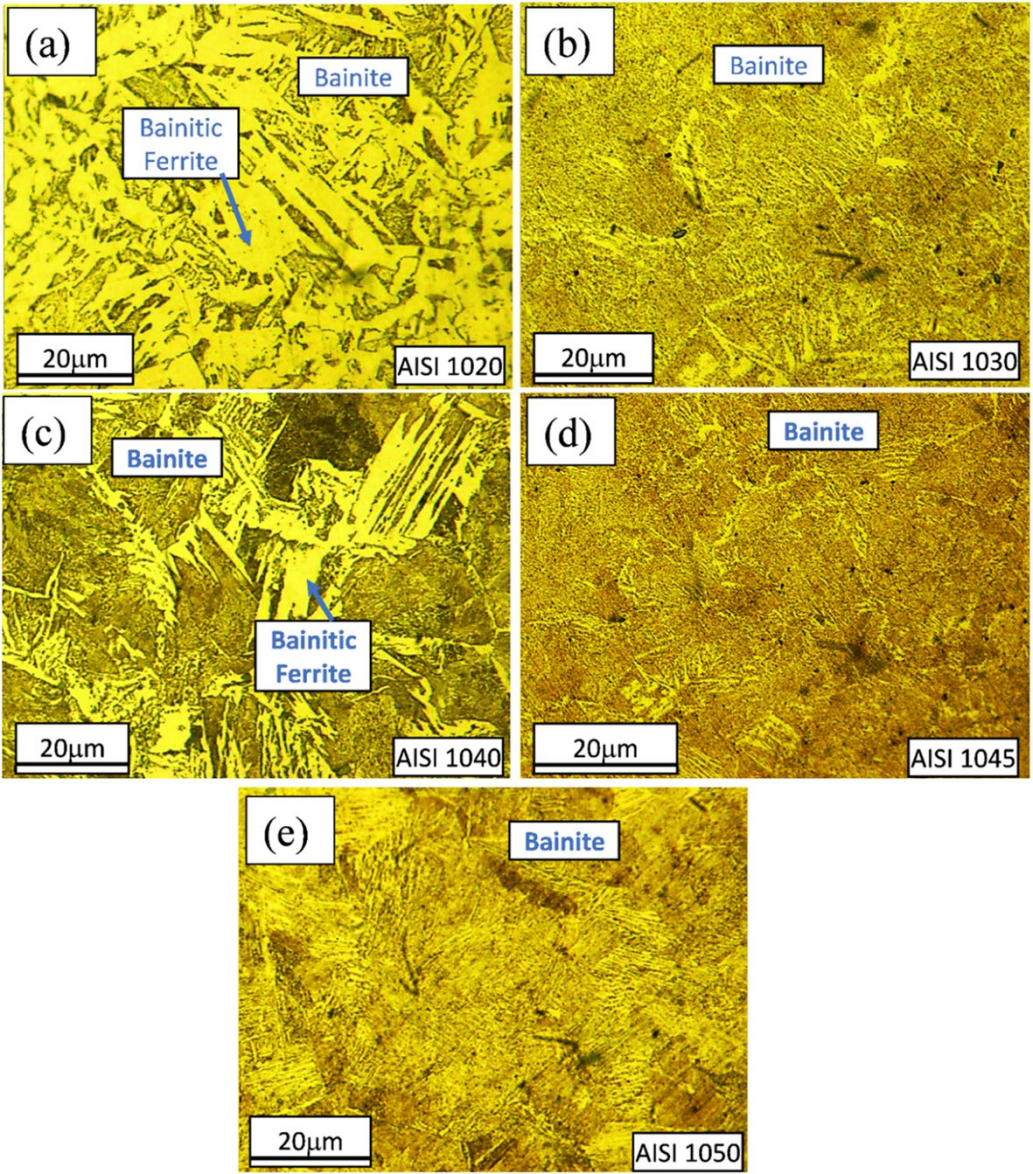
Light optical micrographs showing lower-bainitic microstructures of AISI **a** 1020, **b** 1030, **c** 1040, **d** 1045 and **e** 1050 plain carbon steels showing bainitic microstructure as obtained after austempering

Figures 2, 3, 4 and 5 are showing microstructures of heat-treated plain carbon steels i.e. AISI 1020, 1030, 1040, 1045, and 1050 having carbon content in the range of 0.19–0.54 wt.%. Heat-treatment processes; annealing, normalizing, quenching, and austempering of plain carbon steels resulted in the formation of coarse ferrite-pearlite, fine ferrite-pearlite, martensite, and bainite phases in the microstructures respectively. Microstructures of all the annealed plain carbon steels (Fig. 2) comprised of coarse lamellar pearlite phase in the matrix of ferrite. The annealing process involves slow furnace cooling which provides sufficient time for recrystallization and grain growth, resulting in the coarse-grained microstructure. Due to slow and extended cooling within the furnace, the growth of ferrite is increased within the cementite plates resulting in the formation of coarse pearlite. The lower carbon content (0.19 wt.%) of AISI 1020 steel, resulted in a smaller volume fraction of pearlite in the matrix of ferrite (Fig. 2a). Since the carbon content (0.34 wt.%) of AISI 1030 steel is greater than AISI 1020 steel, it has a large volume fraction of pearlite (Fig. 2b). Similarly, AISI 1040, 1045, and 1050 steels are comprised of increasing pearlite volume fractions in the microstructures (Fig. 2c-e) due to an increase in carbon contents ranging from 0.37–0.54 wt.%.

Relatively fine pearlite in the fine ferritic matrix is observed in the microstructures of normalized plain carbon steels (Fig. 3).

After quenching, packets and blocks of lath martensite (Fig. 4) are visible in all plain carbon steels. It is observed that with an increase in carbon content from 0.37–0.54 wt.%, the martensite laths become finer as reported previously (Schmitt and Horstemeier 2006).

Austempering heat-treatment resulted in the formation of lower bainite (Fig. 5). Due to the higher carbide formation, volume fractions of bainitic ferrite (BF) decreased as an increase in carbon content. The microstructure (Fig. 5b) shows bainitic ferrite, which has Widmanstätten side-plate morphology.

### Micro Vickers hardness

**Fig. 6:**
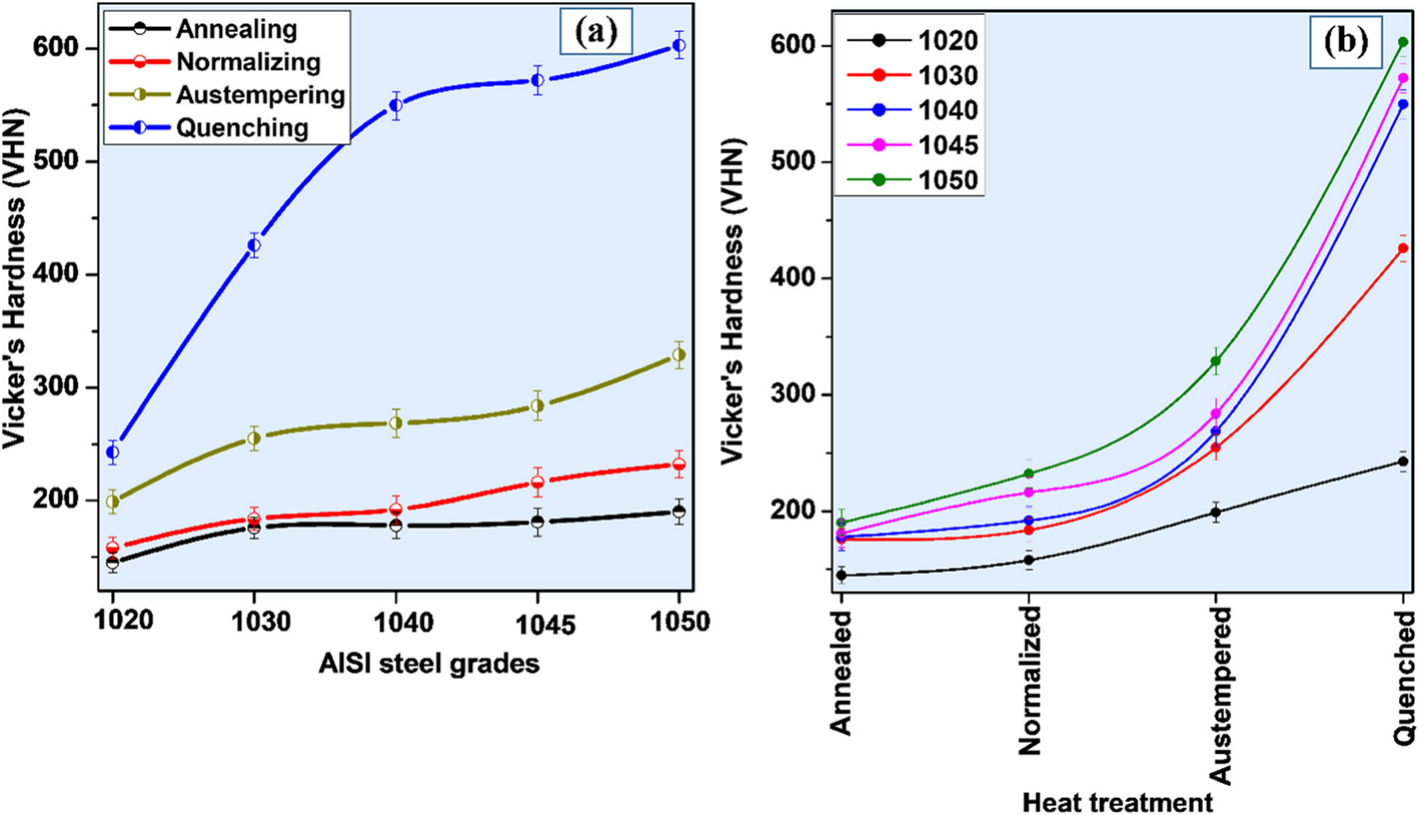
Micro Vickers hardness profile of all plain carbon steels after various heat-treatments

Figure 6a and b shows the micro-Vickers hardness results of plain carbon steels obtained after various heat treatments i.e. annealing, normalizing, quenching, and austempering. Microstructures of annealed plain carbon steels comprised of coarse pearlite phase in the matrix of ferrite. The presence of ferrite in the microstructure of annealed samples caused low hardness values. But with an increase in carbon contents from 0.19 to 0.54 wt.% the amount of cementite phase increased, resulting in a gradual increase in hardness.

A gradual increase in hardness values was observed with an increase in carbon contents. Bainite formed by austempering has been reported to be a comparatively hard phase than ferrite and pearlite. Therefore, the hardness of austempered plain carbon steel was higher than normalized and annealed samples. Supersaturated lath martensite resulted from quenching and possessed the highest hardness among all.

### Electrochemical properties

**Fig. 7:**
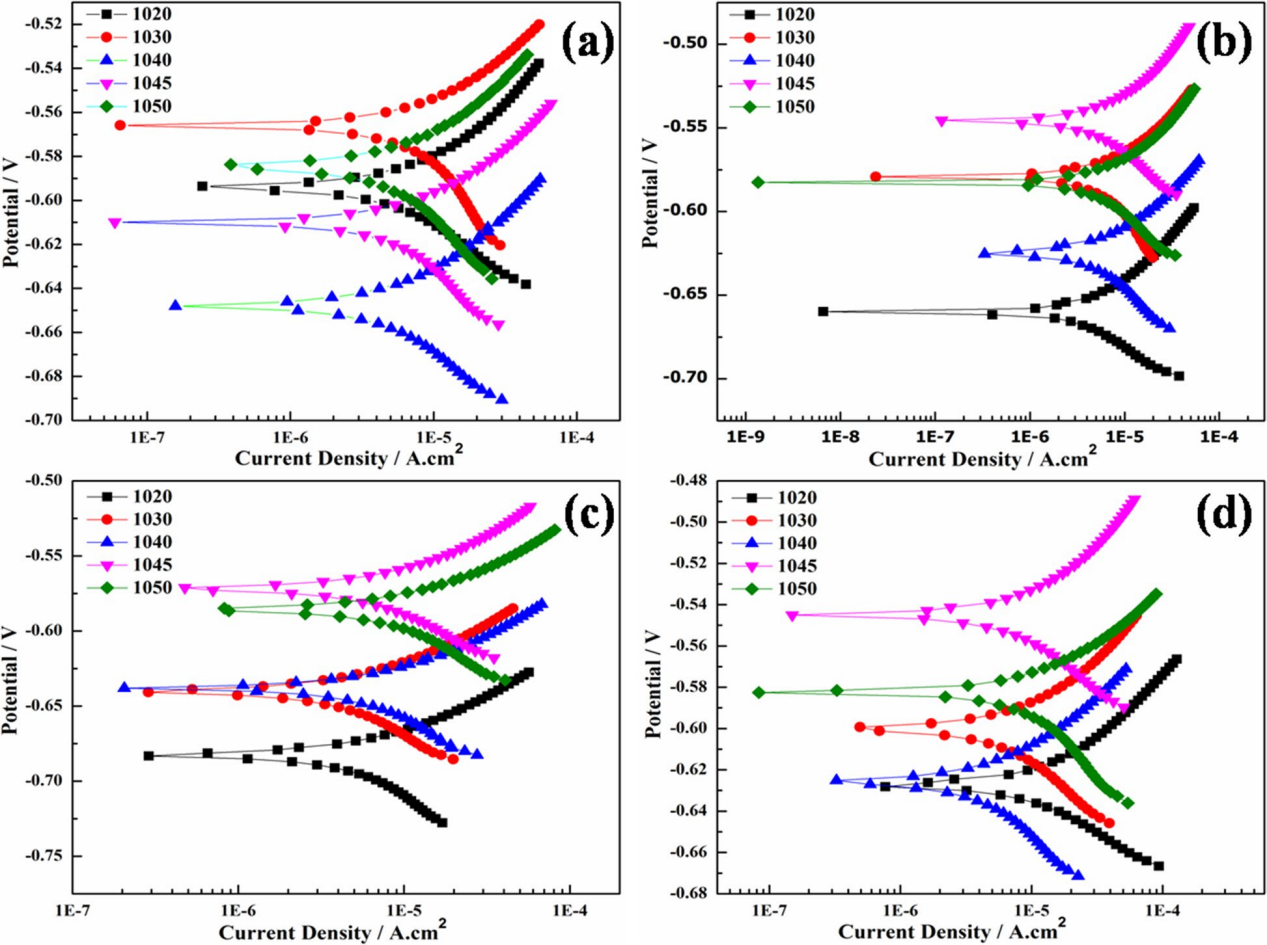
Tafel scan plots of plain carbon steels **a** coarse ferrite-pearlite, **b** fine ferrite-pearlite, **c** lath martensite, and **d** bainite microstructures

Figure 7 is showing Tafel polarization scans of heat-treated plain carbon steels in 3.5% NaCl solution. All the samples are polarized in the ±50 mV potential range with respect to their open circuit potential.

The kinetic parameters like anodic (β_a_) and cathodic (β_c_) slopes, corrosion current densities (i_corr_), corrosion potentials (E_corr_), and corrosion rates were calculated by Tafel fit, with the help of Echem Analyst software (version 5.62).

Table 3 is showing the calculated values of polarization curves. Localized galvanic corrosion cells were formed in seawater due to various concentrations and morphologies of ferrite, pearlite, and cementite. The relatively active potential of the ferrite phase compared with the pearlite phase may have promoted its preferential dissolution. The corrosion of plain carbon steel starts with the oxidation of the ferrite phase due to the occurrence of reaction mentioned in eq. 2 (Roberge 2012).

2 $$Fe\to {Fe}^{2+}+2{e}^{-}$$

**Table 3:** Kinetic parameters calculated from Tafel scan of all heat-treated plain carbon steels

AISI Steel Grades	β_a_ (mV-decade^− 1^)	β_c_ (mV-decade^− 1^)	i_corr_ (μA-cm^− 2^)	E_corr_ (mV)	Corrosion Rate (mpy)
Annealing					
1020	36.30	42.60	5.200	- 594.0	2.378
1030	33.20	51.90	6.230	- 566.0	2.845
1040	85.10	142.0	12.00	- 648.0	5.500
1045	84.20	113.1	16.30	- 545.0	7.352
1050	99.70	1331	21.20	- 585.0	9.666
Normalizing					
1020	34.80	36.60	3.440	- 660.0	1.571
1030	41.60	73.60	6.330	- 579.0	2.892
1040	58.50	109.9	8.960	- 625.0	4.095
1045	86.40	138.5	13.20	- 546.0	5.940
1050	78.30	162.3	13.90	- 583.0	6.362
Quenching					
1020	42.00	77.00	5.640	- 683.0	2.576
1030	67.40	147.0	8.290	- 640.0	3.786
1040	67.70	173.1	12.50	- 638.0	5.725
1045	79.20	154.5	14.90	- 572.0	6.719
1050	66.80	148.4	17.30	- 586.0	7.889
Austempering					
1020	21.80	25.10	5.870	- 627.0	2.681
1030	69.10	136.6	13.70	- 600.0	6.275
1040	71.90	128.2	8.200	- 626.0	7.747
1045	81.60	549.1	18.60	- 610.0	8.381
1050	80.00	296.5	26.30	- 582.0	12.12

On the cathodic site, the reaction (eq. 3) is.

3 $$2{H}_2O+2{e}^{-}\to {H}_2+2{OH}^{-}$$

So, the overall reaction (eq. 4) will be.

4 $${Fe}^{+2}+2{H}_2O\to Fe{(OH)}_2+2{H}^{+}$$

**Fig. 8:**
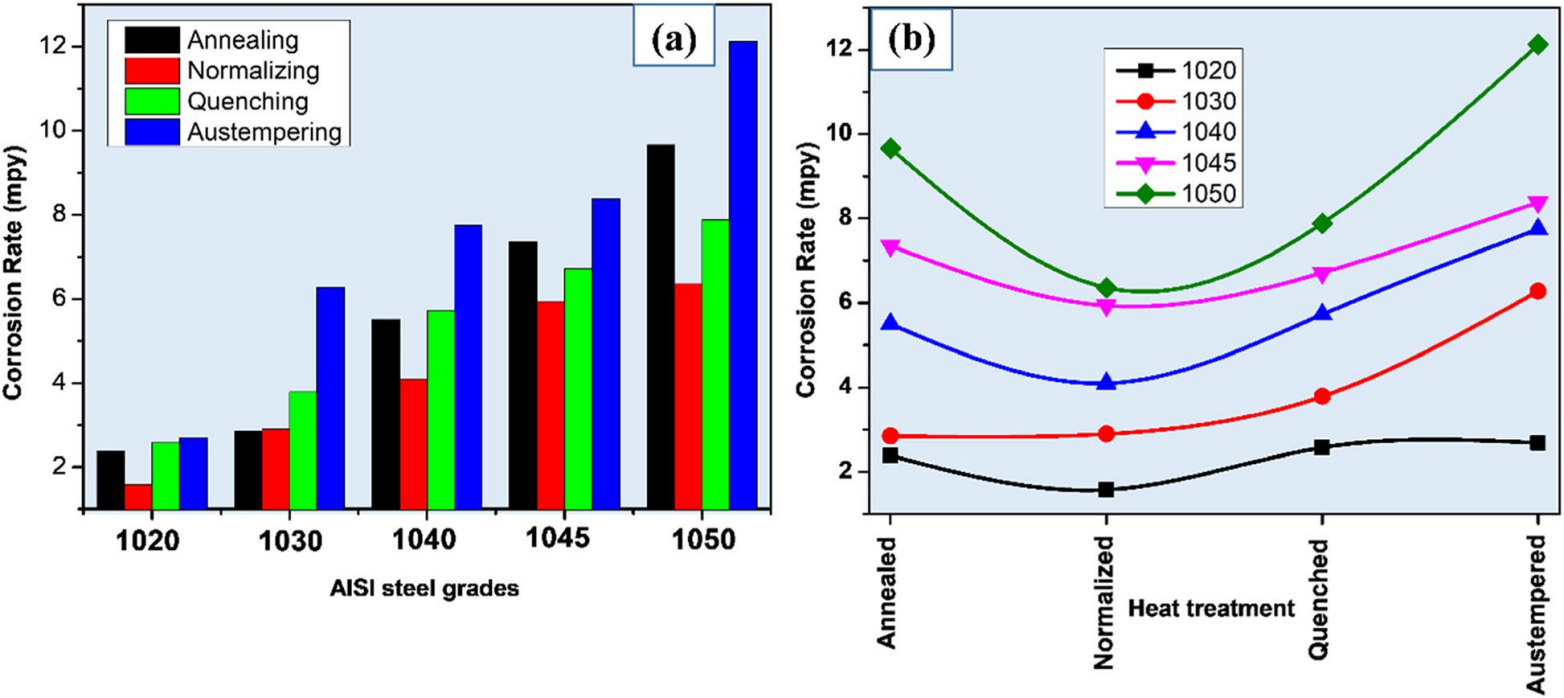
Graphical representation of **a** carbon doping effect and, **b** Heat treatment effect on corrosion rate of different AISI steel samples after electrochemical testing in 3.5% NaCl solution

Hence the ferrite phase acts as anode and cementite as cathode which will further enhance corrosion of plain carbon steels (Fauzi et al. 2019). As shown in Fig. 8a with an increase in carbon concentration from 0.19 to 0.54 wt.% in the plain carbon steel (annealed condition), the corrosion rate also increased from 2.378 to 9.666 mpy due to an increase in the pearlite phase providing more sites for active cell formation.

Decreased corrosion rate after normalizing might be due to the fine grain-sized microstructure compared to annealed samples (Fig. 8b). After normalizing, the highest corrosion rate (6.362 mpy) was exhibited by AISI 1050 steel (0.54 wt.% C) in 3.5% NaCl solution. It has been reported that in 0.5 M NaCl solution, quenched samples exhibited better corrosion resistance than the annealed samples due to less localized galvanic cells (Yong et al. n.d.) formation.

In quenched microstructure carbon is entrapped in BCT crystal structure, resulting in the uniform distribution of carbon in the matrix. Hence, the martensite phase behaves as noble and acts as the cathodic phase while the Widmanstatten ferrite at grain boundaries acts as an anode. Increasing the carbon concentrations from 0.19 to 0.54 wt%, the corrosion rate also increased from 2.576 to 7.889 mpy due to the formation of high carbon martensite. The martensite has more corrosion resistance in comparison to ferrite-pearlite phases. This infers that uniform distribution of carbon in the matrix enhances corrosion resistance. However, the corrosion rate of quenched samples was more than normalized samples as the martensitic microstructure showed less resistance to electron charging and discharging, that made it susceptible to dissolve more as compared to fine ferrite-pearlite (Katiyar et al. 2019).

In the case of austempering, bainitic microstructure showed that increment in the carbon concentration resulted in enhancement of corrosion rate. The corrosion rate is increased from 2.681 to 12.12 mpy with the increase of carbon concentration (Guo et al. 2008b) from 0.19 to 0.54 wt%. Among all microstructures, the highest corrosion rate is observed in the bainitic microstructure due to more active corrosion cells.

### Corrosion morphology

**Fig. 9:**
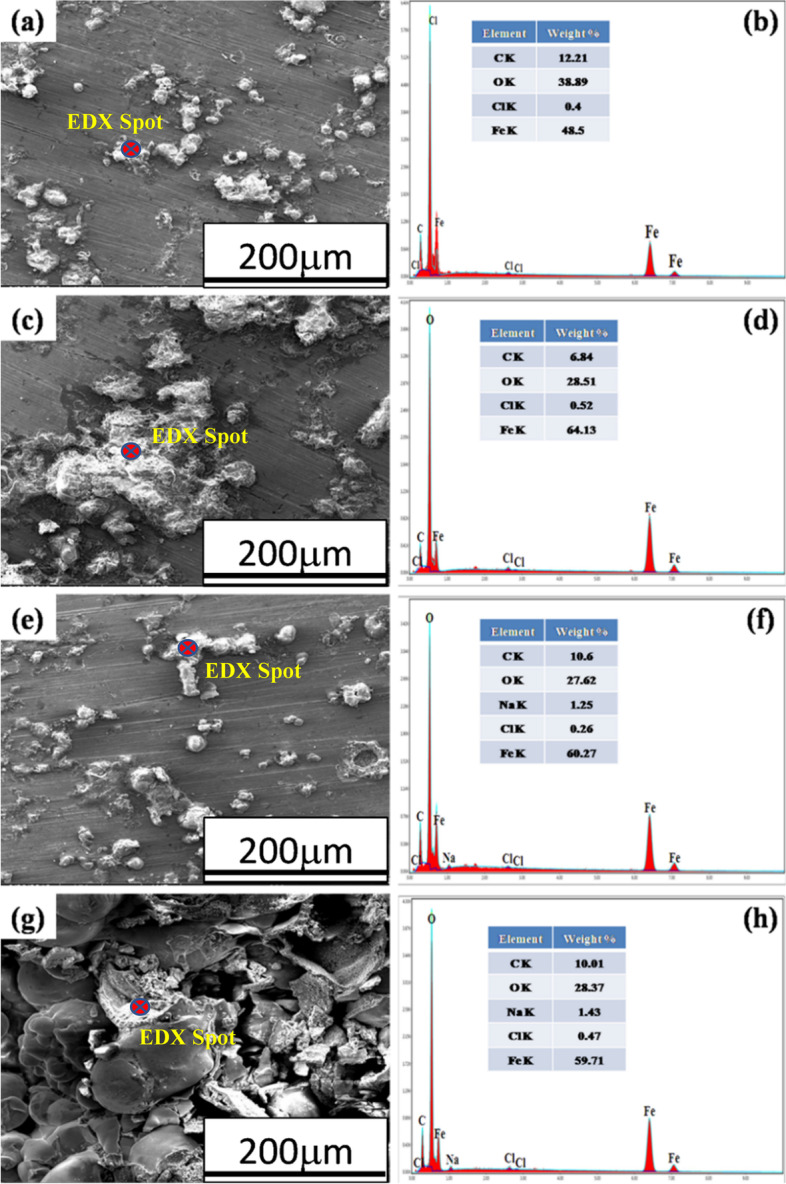
SEM-EDS spot analyses of corrosion products of **a**, **b** annealed, **c**, **d** normalized, **e**, **f** quenched, and **g**, **h** austempered, AISI 1030 steel samples

**Fig. 10:**
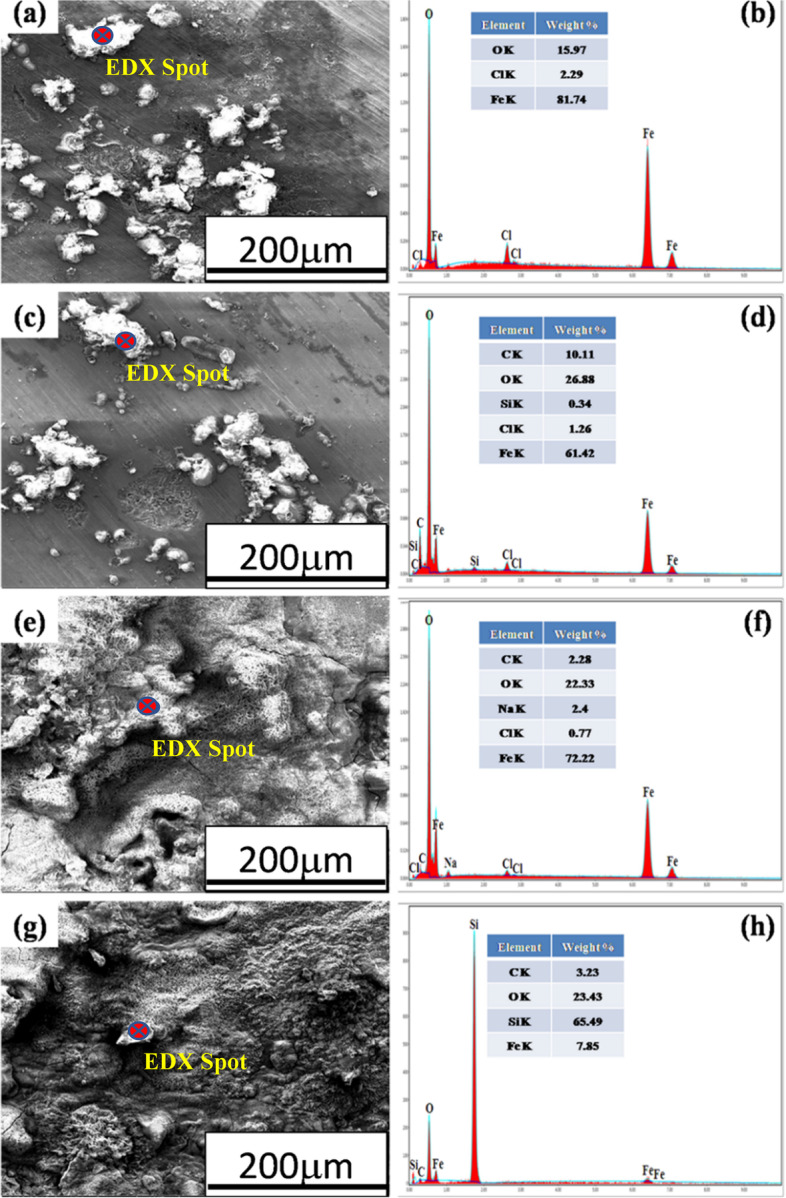
SEM-EDS spot analysis of corrosion products of **a**,**b** annealed, **c**,**d** normalized, **e**,**f** quenched, and **g**,**h** austempered AISI 1050 steel samples

**Fig. 11:**
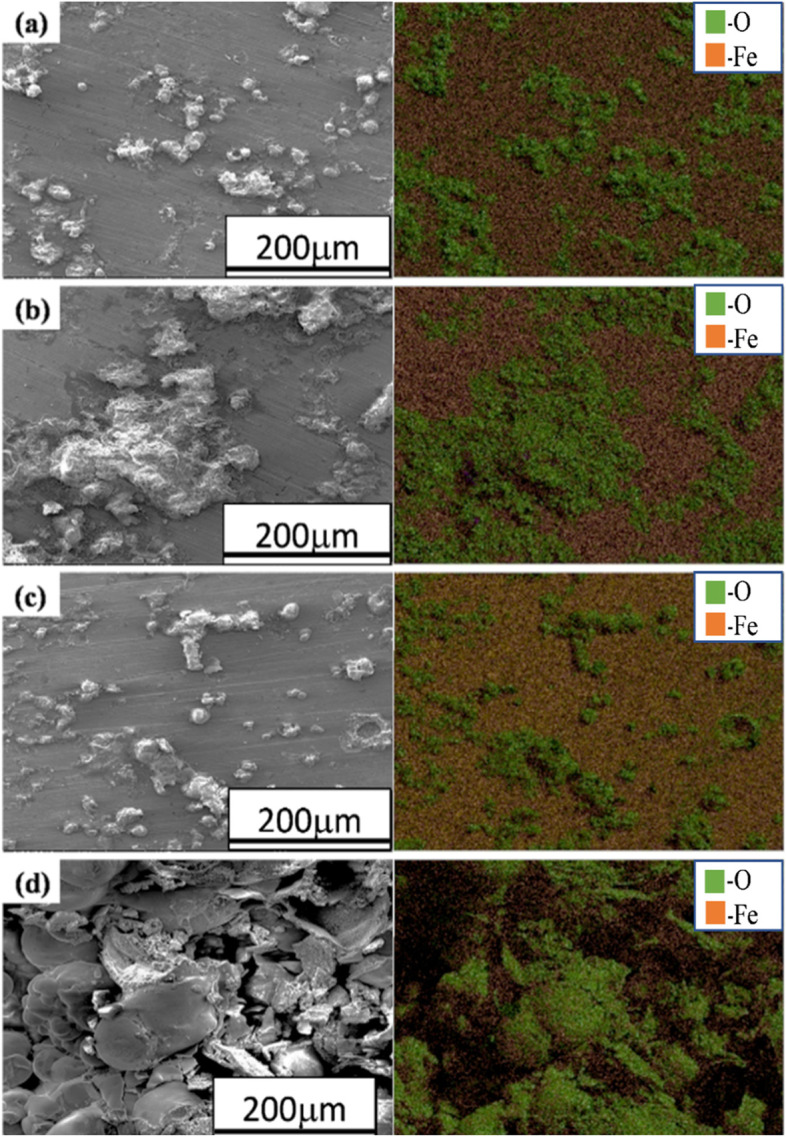
SEM-EDS elemental maps of the corroded surface of **a** annealed, **b** normalized, **c** quenched, and **d** austempered AISI 1030 steel specimens after electrochemical testing in 3.5% NaCl solution

**Fig. 12:**
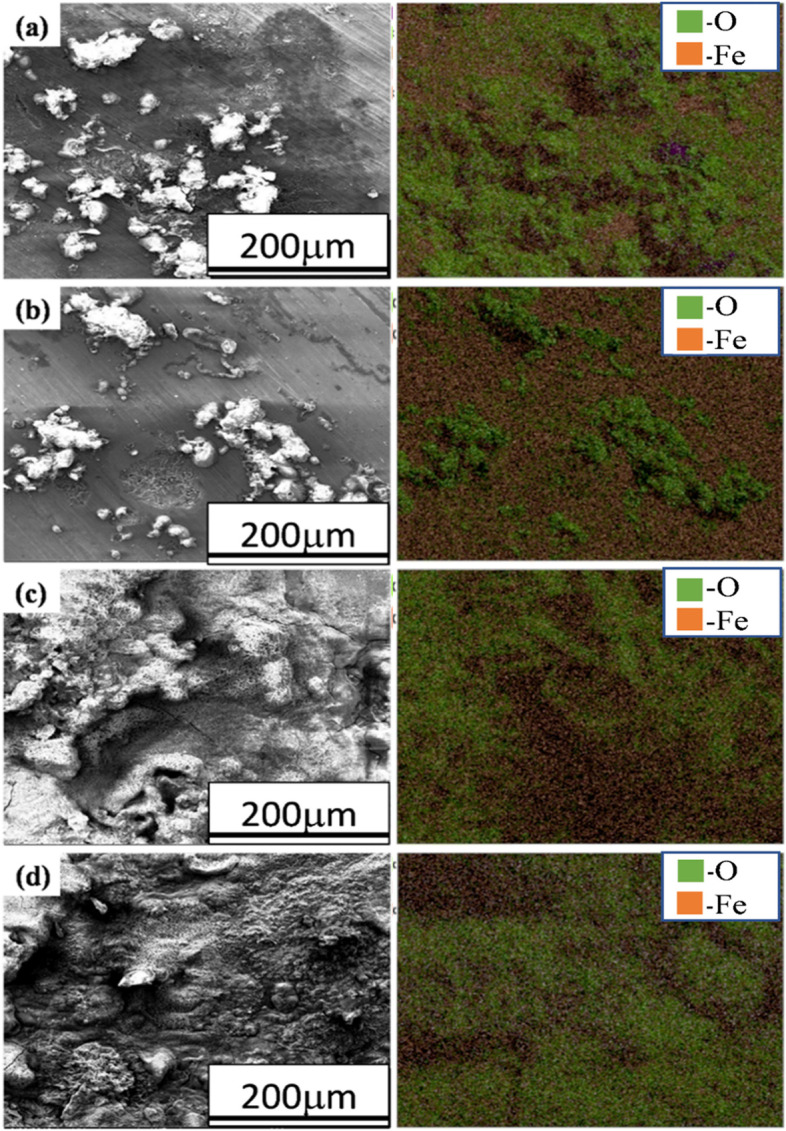
SEM-EDS elemental maps of corroded surface of **a** annealed, **b** normalized, **c** quenched, and **d** austempered AISI 1050 steel specimens after electrochemical testing in 3.5% NaCl solution

SEM micrographs and EDS spectra of heat-treated AISI 1030 and 1050 steel obtained after corrosion testing in 3.5% NaCl solution are shown in Figs. 9 and 10. The SEM-EDS elemental maps of the corroded surface was used to determine the elemental distribution of the corrosion product (Figs. 11 and 12).

The ball shape morphology (Fig. 9) of corrosion products was observed on the surfaces of annealed, quenched, and austempered samples of AISI 1030 steel after corrosion testing which might be goethite, as reported by C. Yong et al. (Kang et al. 2014). While the normalized steel sample revealed flake-like morphology (Fig. 9c) of corrosion product.

The relatively large ball shape morphology (Fig. 9g) of corrosion products was visible on the austempered AISI 1030 sample resulting in a large corrosion rate as revealed in Table 3.

In the case of AISI 1050 steel, again ball shape morphology (Fig. 10) of corrosion products is observed on surfaces of annealed and normalized samples. While on the surfaces of quenched and austempered samples more uniform porous corrosion deposits (Fig. 10e and g) are observed. The elemental maps (Fig. 11) of annealed, normalized, and quenched AISI 1030 steel samples exhibited the localized corrosion attack, whereas, in austempered AISI 1030 steel, uniform corrosion occurred.

When iron ions react with chlorine or oxygen or any other such anions, they tend to form metallic oxides, hydro-oxides, or even chlorides and then deposit as particles. These oxides or hydro-oxides accumulate and may deposit in the areas called crevices and then further trigger corrosion. High percentages of oxygen in the elemental maps indicates high corrosion rates similar to reported works earlier (Antunes et al. 2003; Gheno et al. 2011; Maslar et al. 2001; Yang et al. 2012a; Sarin et al. 2004a).

In the case of AISI 1050, annealed and normalized samples (Fig. 12a and b) underwent localized corrosion attack resulting in the ball-like morphology of corrosion products. In Figs. 11 and 12, elemental maps show the occurrence of Fe and O in almost every map nd these two elements are the dominant part of corrosion scales (Sarin et al. 2004b; Schock et al. 2008; Wang et al. 2014; Li et al. 2016a; Yang et al. 2012b; Tang et al. 2006; Tuovinen et al. 1980).

Magnetite (Fe_3_O_4_) (Lee 2014) along with goethite (α-FeOOH), and lepidocrocite (γ-FeOOH) (Xiao et al. 2021) were observed in the corrosion deposits mainly however small fractions of hematite (Fe_2_O_3_) (Kim et al. 2005b; Lee et al. 2002) calcite (CaCO_3_) and green rusts (hydrated ferrous-ferric compounds containing CO_3_^2−^, Cl^−^ or SO_4_^− 2^) were also observed (Lytle et al. 2004; Gerke et al. 2008; Peng et al. 2010; Li et al. 2016b).

**Fig. 13:**
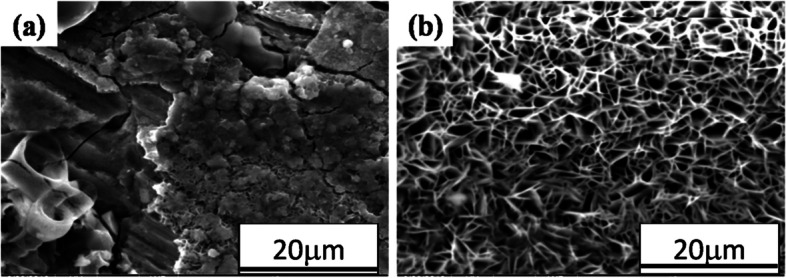
Corrosion deposit morphology of **a** normalized and **b** austempered AISI 1050 steel samples after electrochemical testing in 3.5% NaCl solution

Figure 13 shows higher magnification morphologies of corrosion deposits after corrosion testing of AISI 1050 steel of annealed and austempered microstructures.

The porous/spongy corrosion deposits observed in austempered AISI 1050 steel (Fig. 13b) seemed to be the main cause of the high corrosion rate. Corrosion causes porous morphology which increases the ingress of electrolyte and results in the accelerated dissolution process. However, it seems that the solid morphology of the corrosion deposit leads to the lowest corrosion rate of the normalized AISI 1050 steel (Fig. 13a). Since the solid corrosion product with hairline cracks has a very low ability to retain the electrolyte, results in the lower corrosion rate.

## Conclusions

The different microstructures have been developed by applying heat-treatment process on AISI steel and their microstructure, hardness and corrosion properties have been investigated in detail. Following are the conclusions of the present work;

- The hardness of the plain carbon steel is directly proportional to the carbon contents, AISI1050 shows the maximum whereas AISI1020 minimum. Along with this quenched samples showed the highest, austempered moderate while normalized and annealed exhibited the lowest hardness values.
- With an increment in the carbon concentration, the corrosion rate of the plain carbon steels increases in all the compositions in the order of AISI1050 > AISI1045 > AISI1040 > AISI1030 > AISI1020.
- The bainitic microstructure shows the highest corrosion rate owing to the inhomogeneous distribution of the carbon in the ferrite matrix in the form of carbides whereas the normalized microstructure shows the lowest corrosion rate owing to the relatively homogeneous distribution of the carbon in the ferrite matrix in the form of carbides.
- Porous corrosion deposits revealed a high corrosion rate as compared to solid deposits.

## Data Availability

All relevant data have been included in the manuscript itself.

## Abbreviations

SEM: Scanning Electron Microscope
AISI: American Iron and Steel Institute
AI: Artificial Intelligence
mpy: Mills Per Year
EDS: Energy Dispersive X-ray Spectroscopy
