# Correction: Microstructural, mechanical, and electrochemical analysis of carbon doped AISI carbon steels

**DOI:** 10.1186/s42649-024-00102-2

**Published:** 2024-10-11

**Authors:** Muhammad Ishtiaq, Aqil Inam, Saurabh Tiwari, Jae Bok Seol

**Affiliations:** 1 Department of Materials Engineering and Convergence Technology, Gyeongsang National University (GNU), Jinju, 52828 Republic of Korea; 2 Department of Materials Engineering and Convergence Technology, Center for K-metal, Gyeongsang National University (GNU), Jinju, 52828 South Korea; 3 Institute of Metallurgy and Materials Engineering, University of the Punjab, Lahore, Pakistan

**Correction: Appl. Microsc 52, 10 (2022)**

10.1186/s42649-022-00079-w

Following publication of the original article (Ishtiaq et al. 2022), the authors would like to replace the first listed Funding statement from:

**Current funding**

J.B.S. acknowledges financial support from the National Research Foundation of Korea (NRF) grant funded by the Korea Government (No. 2021R1A2C4002622)

**New funding**

This work was supported by the research grant of the Gyeongsang National University in 2022.

This correction does not affect the overall result or conclusion of the article. The original article (Ishtiaq et al. 2022) has been corrected.
